# Editorial: Advances in genomic medicine and gynecological sciences

**DOI:** 10.3389/fonc.2026.1776721

**Published:** 2026-01-20

**Authors:** Andrea Giannini

**Affiliations:** Unit of Gynecology, Department of Surgical and Medical Sciences and Translational Medicine, Sant’Andrea Hospital, Sapienza University of Rome, Rome, Italy

**Keywords:** artificial intelligence, biomarkers, genomic medicine, gynecological oncology, molecular profiling, precision medicine

Genomic medicine has profoundly transformed gynecological knowledge, principally within oncology, by enabling a more sophisticated understanding of tumor biology, interpatient heterogeneity and mechanisms of disease progression and treatment resistance. High-throughput sequencing technologies, molecular profiling and integrative analytical approaches have contributed to a paradigmatic shift from classic clinicopathological classifications to biologically determined and personalized management strategies. Despite these advances, the clinical translation of genomic innovations remains complex, requiring strong validation, interdisciplinary integration and careful consideration of ethical, logistical, and healthcare system–related challenges.

The main aim of this Research Topic was to explore recent advances in genomic medicine applied to gynecological cancers, with a specific focus on prognostic stratification, biomarker discovery and the integration of molecular and clinical data into tailored oncological care. The studies included in this Research Topic highlight how genomic and biomarker-driven approaches are remodeling diagnostic accuracy, therapeutic decision-making and outcome prediction throughout different gynecological malignancies. A total of five high-quality papers were published within this Research Topic, including systematic reviews, original research articles and narrative reviews, emphasizing the growing relevance of translational genomics in gynecological oncology.

Within this Research Topic, Chen et al. provide a synthesis of the evidence linking DNA repair variation to gynecological cancer risk. Their meta-analysis evaluates ERCC2 polymorphisms (Lys751Gln, Asp312Asn, and Arg156Arg) and reports that Lys751Gln is associated with increased susceptibility to gynecological tumors, with the strongest signal for ovarian cancer, whereas Asp312Asn and Arg156Arg do not appear to increase overall risk; notably, a recessive Asp312Asn model may even be protective for cervical cancer in Asian populations. These findings reinforce the broader concept that inherited variation in DNA repair pathways can influence tumor predisposition and may also interact with treatment response, particularly where platinum sensitivity and resistance are essential therapeutic issues.

Moving from genetic susceptibility toward clinically actionable monitoring, Wang et al. report a persistent challenge in locally advanced cervical cancer: the heterogeneity of outcomes after chemoradiotherapy. In a prospective cohort of FIGO IB–IVA patients treated with standard chemoradiotherapy and brachytherapy, they propose a multi-biomarker panel incorporating inflammatory markers and tumor-associated proteins (including IL-6, CRP, SCC-Ag, CA125, and hematologic ratios) to support early response stratification and prognostication. Their work aligns with current efforts to develop minimally invasive biomarker approaches that can balance imaging and clinical staging, with the practical purpose of identifying patients who may benefit from intensified surveillance or tailored therapeutic strategies.

The Research Topic also emphasizes how computational methods grounded in clinical application can help bridge the “bench-to-bedside” gap. Molefi et al. review the growing role of artificial intelligence in type II endometrial cancer, framing AI as a tool to improve diagnosis, prognostic assessment and treatment planning in a high-mortality disease subtype frequently managed similarly to less aggressive type I tumors.

Importantly, the review highlights global trends while also addressing African contexts, underscoring that innovation in precision oncology must be accompanied by implementation pathways that reduce disparities in access to advanced diagnostics and targeted care.

Prognostic refinement is a recurring theme across gynecological malignancies, and Chen et al. contribute a systematic review and meta-analysis focused on lymph node ratio (LNR) as a prognostic indicator. By combining data across gynecological cancers, they report an association between higher LNR and worse oncological outcomes. Beyond confirming prognostic relevance, their analysis raises clinically practical questions illustrating how quantitative pathology-derived metrics can be integrated into modern risk stratification frameworks.

Finally, Shi and Chen present a helpful case report that highlights diagnostic complexity at the interface of morphology, immunophenotype and clinical context. They describe a primary cervical gestational choriocarcinoma that can mimic squamous cell carcinoma, emphasizing how careful clinicopathologic correlation, including hCG assessment and appropriate immunohistochemistry, can prevent misclassification and enable effective systemic therapy.

While case reports do not aim to provide generalizable molecular models, this contribution is highly aligned with the Topic’s translational focus: rare entities and diagnostic traps often represent the hardest tests of precision medicine in everyday practice.

These five articles reflect complementary angles of progress in genomic medicine and gynecological sciences: germline variation and cancer susceptibility, biomarker-enabled response prediction and monitoring, AI-supported precision approaches, prognostic refinement through quantitative metrics and diagnostic accuracy in rare but high-stakes clinical scenarios. Collectively, they reinforce a central message of the Research Topic: meaningful advances in gynecological oncology increasingly depend on integrating molecular and computational innovation with rigorous clinical validation, clear diagnostic pathways, and equitable strategies for adoption.

